# Unveiling the associations between conscientiousness and emotional intelligence in Paralympic athletes

**DOI:** 10.3389/fpsyg.2024.1477410

**Published:** 2024-12-04

**Authors:** Murat Sarikabak, Mert Ayranci, Ilimdar Yalcin, Laurentiu-Gabriel Talaghir, Cristina-Corina Bentea

**Affiliations:** ^1^Faculty of Sport Sciences, Bartin University, Bartın, Türkiye; ^2^Faculty of Sport Sciences, Hitit University, Corum, Türkiye; ^3^Faculty of Sport Sciences, Bingöl University, Bingöl, Türkiye; ^4^Faculty of Physical Education and Sport, Dunarea de Jos University of Galati, Galati, Romania

**Keywords:** sports, athletes, Paralympic, emotional intelligence, conscientiousness

## Abstract

This study investigates the association between conscientiousness and emotional intelligence (EI) in Paralympic athletes. A sample of 274 athletes (190 male, 84 female) was analyzed using the Schutte EI Scale and a Conscientiousness scale from the Big Five Personality Traits. Results indicated a significant positive relationship between EI and conscientiousness, with EI explaining 28% of the variance in conscientiousness (*p* < 0.05). Specifically, optimism and mood regulation demonstrated a high positive correlation with conscientiousness (r = 0.501), while assessing feelings showed a moderate positive correlation (r = 0.391), and using feelings revealed a low positive correlation (r = 0.120). Gender-based analysis revealed that female athletes scored significantly higher in emotional usage. These findings suggest that promoting EI in Paralympic athletes could enhance conscientiousness, potentially benefiting their resilience and psychological stability. Future research should explore longitudinal designs to examine these associations further.

## 1 Introduction

Paralympic athletes not only need to overcome physical barriers but also require high-level psychological skills (Pankowiak et al., 2023). These athletes strive to overcome challenges effectively by utilizing psychological skills such as resilience, motivation, focus, and coping with stress (Olive et al., 2021). Despite facing challenges, Paralympic athletes often stand out for their ability to display a positive mental attitude, turning difficulties into opportunities (Swartz et al., 2019; Henriksen et al., 2020; Lüdi et al., 2023). Additionally, the tendency of Paralympic athletes to be part of a distinct solidarity and feel a sense of belonging to a community that serves as a strong motivational source can enhance their psychological resilience (Öner, 2023; Trainor et al., 2023). Moreover, the support within the team, the sense of collaboration, and solidarity can contribute to maintaining a positive mental state for the athletes.

Most Paralympic athletes possess an extraordinary source of motivation by combining their personal experiences in overcoming obstacles with a strong desire to achieve their goals (Rosa et al., 2020; Marques and Alves, 2021). This motivation enables them to approach challenging training sessions, competitions, and life in general with a more positive outlook, consequently enhancing their psychological resilience (Martin, 2017; Hogg, 2018; Mira et al., 2023). As a result, the psychological skills of Paralympic athletes not only enable them to overcome obstacles but also facilitate high-level performance. Therefore, possessing strong psychological skills may help elevate Paralympic athletes beyond their current levels. Undoubtedly, one of the most crucial relationships in dynamics athletes establish with their social environment, coaches, and teammates is emotional intelligence (Laborde et al., 2016; Zajonz et al., 2024). Emotional intelligence (EI) can be defined as an individual’s awareness of their own emotional state, the ability to assess the emotions of others, the capacity for empathy, and the skill to manage emotions rationally and effectively (Goleman, 2017).

EI plays a significant role within the realm of sports. Athletes can enhance their ability to cope with stress, demonstrate empathy within the team, and maintain emotional balance by utilizing their EI (Wagstaff, 2014). Rigorous training sessions and competitions demand athletes to remain emotionally resilient, potentially leading to an enrichment in EI. Sport can strengthen emotional intelligence through the ability to strike a balance between achievements and failures, thereby assisting athletes in effectively performing even in stressful moments (Khanin, 2000; Friesen et al., 2020). Regular participation in training requires consistent effort to reach established goals, fostering an individual’s self-discipline (Piepiora, 2021; Kusumawardhanny et al., 2021). Consequently, the positive impact of sports on an individual’s personality development and EI can contribute to comprehensive improvement not only in physical health but also in enhancing overall life quality.

The relationship between EI and conscientiousness reflects a complex connection, where an individual’s ability to understand, manage, and effectively utilize their emotional states is intricately linked to conscientiousness (Szcześniak et al., 2020). Although EI has been widely studied in general sports psychology, research on the unique psychological challenges faced by Paralympic athletes remains limited. In this context, understanding the role of conscientiousness within EI for this population may offer valuable insights into their psychological resilience. EI encompasses the ability to recognize, express, understand, and manage emotions (Goleman, 2017), while conscientiousness involves the skills of control, organization, and stability to achieve long-term goals (Somer et al., 2002).

As an individual enhances their EI, they better understand and manage their emotional responses. This is directly associated with conscientiousness because emotional intelligence can increase one’s ability to resist instant gratification and impulses. For instance, an individual with high emotional intelligence can control their emotional reactions when faced with emotionally challenging situations and can devise a more effective strategy to resolve the situation. This scenario involves conscientiousness working harmoniously, allowing a focus on long-term goals and the ability to defer when necessary.

Conscientiousness is associated with the insight and control provided by emotional intelligence (Tok, 2008; Van der Linden et al., 2017). Individuals with increased emotional intelligence can more effectively apply the conscientiousness needed to stay committed to their goals even in emotionally challenging moments (Di Fabio and Saklofske, 2018). Therefore, this mutual interaction between emotional intelligence and conscientiousness is indicative of how EI is related to enhanced conscientiousness. Consequently, it enables the individual to be more successful both emotionally and behaviorally. Thus, the main objective of this study was to investigate the predictive role of the concept of conscientiousness in the emotional intelligence levels of Paralympic athletes. The modern integration of the EI concept across diverse academic disciplines, including Sports, (Laborde et al., 2018), Health (Schutte et al., 2007), Tourism (Borges et al., 2022), emphasizes the significance of EI. Conscientiousness underscores athletes’ prospective accomplishments by accentuating their capacity to regulate their behavior, maintain goal-oriented focus, and assume accountability. Collectively, EI and conscientiousness can empower an individual to more effectively cope with challenges in both social relationships and the sporting environment, enhancing their overall ability to navigate difficulties in life.

*H1. As the conscientiousness levels of Paralympic athletes increase, their optimism and mood regulation skills will also improve.*

*H2. High conscientiousness in Paralympic athletes will have a positive impact on their ability to use emotions.*

*H3. Paralympic athletes with high levels of conscientiousness will show an improvement in their ability to assess emotions.*

*H4. Conscientiousness will positively affect the mood regulation and optimism levels of Paralympic athletes.*

## 2 Methods

### 2.1 The research design

The study employed a relational screening model. In a relational screening model, “research investigates the relationship between two or more variables without intervening variables” (Büyüköztürk et al., 2017).

### 2.2 Participants

In this study, convenience sampling was chosen due to the accessibility of Paralympic athletes and the practical limitations in reaching a broader population through random sampling. The athletes who participated were those readily available during the study period, specifically those attending championships between 2021 and 2022. The inclusion criteria for the study included being a licensed Paralympic athlete who actively participated in international competitions during this time and who voluntarily agreed to participate in the study. The exclusion criteria included athletes who were not actively participating in competitions, those without a license, or those who did not consent to participate. While convenience sampling has limitations regarding the generalizability of results, it was deemed appropriate for the purposes of this exploratory study given the time and resource constraints (Creswell, 2014).

The research population consists of athletes actively participating in sports and attending championships between 2021 and 2022. Researchers used established sampling formulas to determine the sample size from the accessible population. One of these formulas, developed by Yazıcıoğlu and Erdoğan (2004), provides a simple and efficient method to calculate an adequate sample size. According to their table, “From a population of 1,000 individuals, selecting a sample with a margin of error (d) of ±0.05, taking *p* = 0.8 and q = 0.2, 198 individuals are sufficient for the sample.” Based on this method, the sample for this research included 274 disabled athletes who were selected using the convenience sampling method.

All participants in the study are currently continuing their professional athletic careers. The participants’ ages ranged from 18 to 48 years, and their athletic experience varied between 2 and 20 years. The results of the descriptive statistics for the Paralympic athletes are presented in Table 1. According to the analysis, there were 190 male and 84 female athletes, with 225 athletes having physical disabilities, 21 athletes with visual impairments, and 28 athletes with hearing impairments. Moreover, 142 athletes had achieved results in international competitions, while 132 athletes had no international achievements.

**Table 1:** Descriptive statistics of Paralympic athletes.

Variables	Groups	*F*	%
Gender	Male	190	69.3
	Female	84	30.7
Disability Status	Physically Disabled	225	82.1
	Visually Impaired	21	7.7
	Deaf Impaired	28	10.2
Do You Have Medals at International Competitions?	Yes	142	51.8
	No	132	48.2
	Total	274	100.0

### 2.3 Data collection tools

During the data collection process, necessary assistance was provided to visually impaired and other disabled athletes when completing the questionnaires. For visually impaired participants, the questions were read aloud by a research assistant, and the athletes’ responses were directly recorded by the assistant. For participants with physical disabilities, the questionnaires were made accessible, and for those with hearing impairments, the questions were supplemented with visual materials when necessary. This support was provided in a careful and considerate manner to ensure that participants could provide their responses independently. The validity and accuracy of the data were maintained by ensuring that no interference with participants’ responses occurred.

Various data collection tools were utilized in the study. Initially, researchers employed a “Personal Information Form” that had been developed and validated through expert opinions. After obtaining the necessary permissions from relevant institutions for conducting the research, the “Personal Information Form,” “Schutte Emotional Intelligence Scale,” and “Conscientiousness Scale” were prepared. These forms were made available for athletes to fill out under appropriate conditions. In the form prepared by the researchers, the aim was to gather information about the athletes’ genders, disability statuses, and their rankings in international competitions. The EI scale, originally devised by Schutte et al. (1998), underwent revision by Austin et al. (2004), resulting in a reconfiguration comprising 41 items. Tatar et al. (2011) conducted the Turkish adaptation of the scale, which comprises three sub-dimensions: “Optimism and mood regulation,” “Using feelings,” and “Assessing feelings,” employing a 5-point Likert scale. The analysis yielded Cronbach’s alpha reliability coefficient of 0.85 for the scale. The Big Five Personality Inventory, developed by John et al. (1991) and adapted into Turkish by Somer et al. (2002), was utilized in the study. This adapted inventory comprises five sub-dimensions: extraversion, emotional stability, openness to experience, agreeableness, and conscientiousness, totaling 44 items. The conscientiousness subscale specifically has demonstrated strong reliability and validity across different populations. In our current study, the reliability coefficient (Cronbach’s alpha) for the Conscientiousness Scale was found to be 0.75, indicating acceptable internal consistency for this specific sample.

### 2.4 Statistical analysis

The data collected from Paralympic athletes were coded and transferred to the SPSS 25 software package. Before deciding on the analyses to be applied to the data, a normality test was conducted. Following the normality test, it was observed that the data fell within the range of −2 to +2, suggesting a normal distribution of the data (George and Mallery, 2001). In analyzing the data, descriptive statistics, including frequency and percentage values, were examined, *t*-test and Pearson correlation analysis and simple linear regression analysis were performed. To ensure the assumptions for linear regression were met, we conducted the Durbin-Watson test for independence and the Breusch-Pagan test for homoscedasticity. The Durbin-Watson test yielded a result of 2.1, indicating no autocorrelation, and the Breusch-Pagan test confirmed that the assumption of homoscedasticity was satisfied (*p* = 0.24). Thus, the data met the necessary assumptions for linear regression analysis.

## 3 Results

The results of the analysis based on participants’ gender and achievement status (Do You Have Medals at International Competitions?) are presented in Tables 2, 3.

**Table 2:** Comparison of participants’ emotional intelligence and conscientiousness scores by gender.

Variables	Gender	*n*	x̄	SD	*t*	df	*p*
Optimism/Organizing the mood	Male	190	3.98	0.431	−1.900	272	0.058
	Female	84	4.09	0.457			
Using feelings	Male	190	3.36	0.591	−2.638	272	0.006
	Female	84	3.56	0.525			
Assessing feelings	Male	190	3.80	0.598	−1.206	272	0.229
	Female	84	3.90	0.618			
Conscientiousness	Male	190	4.03	0.644	−0.097	272	0.923
	Female	84	4.04	0.596			

**Table 3:** Comparison of participants’ emotional intelligence and self-discipline scores by achievement status.

Variables	Medals at international competitions?	*n*	x̄	SD	*t*	df	*p*
Optimism/Organizing the mood	Yes	142	4.08	0.424	2.708	272	0.007
	No	132	3.94	0.448			
Using feelings	Yes	142	3.45	0.576	0.756	272	0.450
	No	132	3.39	0.580			
Assessing feelings	Yes	142	3.89	0.630	1.536	272	0.126
	No	132	3.77	0.573			
Conscientiousness	Yes	142	3.83	0.376	2.419	272	0.016
	No	132	3.73	0.388			

Table 2 presents the comparison of participants’ emotional intelligence and conscientiousness scores in terms of the gender variable. The analysis revealed a statistically significant difference in the sub-dimension of emotional utilization (*p* < 0.05). This can be explained by the fact that the average scores of women were higher than those of men. There were no significant differences in other sub-dimensions or in conscientiousness.

The comparison of participants’ emotional intelligence and self-discipline scores by achievement status revealed a significant difference in the Optimism/Organizing the Mood sub-dimension and Conscientiousness (p < 0.05). This can be explained by the higher average scores of those who answered “yes.” No significant differences were observed in the other sub-dimensions.

### 3.1 Hypothesis testing and correlation analysis results

The findings support the hypotheses by indicating positive relationships between conscientiousness and various dimensions of EI. Specifically, conscientiousness showed a strong positive correlation with optimism/mood regulation (r = 0.501, *p* < 0.05), a moderate correlation with assessing feelings (r = 0.391, *p* < 0.05), and a low positive correlation with using feelings (r = 0.120, *p* < 0.05), supporting Hypotheses 1, 2, and 3 (Table 4). Furthermore, regression analysis confirmed Hypothesis 4, revealing that conscientiousness explains 28% of the variance in EI, suggesting that conscientiousness enhances mood regulation and optimism, which are essential for psychological resilience in Paralympic athletes (Table 5).

**Table 4:** The relationship between EI scores and conscientiousness scores of Paralympic athletes.

Variables		Conscientiousness
Optimism/Organizing the mood	r	0.501^**^
	*p*	0.001
Using feelings	r	0.120^*^
	*p*	0.048
Assessing feelings	r	0.391^**^
	*p*	0.001

*p ≤ 0.05; **p ≤ 0.01; *n*: 274.

**Table 5:** Regression analysis results: predicting emotional intelligence dimensions from conscientiousness.

Model	β	Std. Error	β	*t*	*p*
Constant	0.895	0.340	---	2.632	0.009
Optimism/Organizing the mood	0.584	0.082	0.412	7.132	0.000
Using feelings	−0.044	0.069	−0.034	−0.634	0.526
Assessing feelings	0.250	0.068	0.216	3.680	0.000
R = 0.536, R^2^_adj_ = 0.279; F_(3,273)_ = 36.230, *p* = 0.000					

According to the results of multiple linear regression analysis in Table 5, it can be observed that the regression model’s optimism/mood regulation and evaluation of emotions sub-dimensions are statistically significant. When scrutinizing the t-test outcomes for the significance of the regression coefficients, it was found that optimism/mood the mood (β = 0.584; *t* = 7.132; *p* = 0.001) had a significant predictive power on conscientiousness along with the assessing feelings (β = 0.250; *t* = 3.680; *p* = 0.001). It can be stated that 28% of the total variance in conscientiousness is explained by emotional intelligence.

## 4 Discussion

The study investigated the role of conscientiousness in predicting the emotional intelligence levels of Paralympic athletes. The statistical analysis revealed that the variance in the conscientiousness levels of Paralympic athletes accounted for 28% of. In our study, the β = 0.584 value obtained in the optimism/mood regulation subdimension indicates a strong effect size according to Cohen’s (1988) criteria. This finding aligns with other studies demonstrating a strong relationship between conscientiousness and emotional intelligence. For instance, Petrides and Furnham (2001) examined the porsitive relationship between emotional intelligence support conscientiousness, reporting that high levels of emotional intelligence support conscientious traits. The β = 0.250 value observed in the emotion assessments sub-dimension is also consistent with Minbashian et al. (2010) findings, which identified a moderate effect between task-oriented conscientiousness and emotional management skills. Goleman (2017) emphasized the crucial role of emotional intelligence in enhancing conscientiousness, particularly in terms of emotional awareness and self-regulation. Schutte et al. (2007) also reported a strong association between the development of emotional intelligence skills and general personality traits. Similarly, Di Fabio and Saklofske (2018) found that emotional intelligence positively influences resilience and self-control, essential personality traits. Studies on Paralympic athletes have shown that high levels of conscientiousness foster a positive interaction with emotional intelligence in elite athletes (Sadri and Janani, 2015). These findings suggest that conscientiousness has a particularly strong effect on dimensions of emotional intelligence such as optimism and underscore the importance of the interaction between emotional intelligence and conscientiousness for this specific population. Additionally, the correlation analysis indicated a significant relationship between the emotional intelligence sub-dimensions of Paralympic athletes. A literature review highlighted the limited number of studies conducted on Paralympic athletes, underscoring the importance of our research. When examining existing research on athletes, congruent findings with our results were observed in the literature.

This supports our hypothesis that conscientiousness can positively influence emotional intelligence, particularly in how individuals regulate and manage emotions. The findings from both studies strengthen the validity of our results and emphasize the role of emotional intelligence in enhancing the effects of conscientiousness. In addition, our results are consistent with the theoretical framework proposed by Mayer and Salovey (1997), which suggests that personality traits like conscientiousness play a crucial role in emotional functioning. This consistency across studies reinforces the validity of our hypothesis, further supporting the positive relationship between conscientiousness and emotional intelligence. Similarly, Tok (2008) has indicated high correlations between emotional intelligence and personality sub-dimensions. Goleman (2017), considered one of the pioneers of the concept of emotional intelligence, addressed the connection between emotional intelligence and conscientiousness in his integrated model. He emphasized that the synergistic interaction of these two concepts would lead to positive outcomes for individuals. Research in literature has highlighted that the concept of conscientiousness facilitates individuals in planning and goal setting, particularly in the context of learning new subjects or demonstrating performance (Karmakar and Ghosh, 2023; Cleary and Chen, 2009). Grewal et al. (2006) asserted a belief that the concepts of emotional intelligence encompass enhancing emotional awareness, managing these emotions appropriately in relationships, accurately perceiving one’s and others’ situations, controlling emotional responses, and establishing effective dominance over immediate desires. They indicated that this approach is centered around conscientiousness.

The gender differences in emotional intelligence (EI), particularly in emotional utilization, align with previous findings showing that female athletes often score higher in specific EI dimensions. Research suggests that women generally exhibit stronger emotional regulation and cognitive appraisal strategies, likely due to enhanced emotional processing capabilities (Karmakar and Ghosh, 2023; Rodríguez-Romo et al., 2021). These findings are consistent with the higher emotional utilization scores observed in female Paralympic athletes in this study. The significant relationship between achievement status and both optimism/organizing the mood and conscientiousness supports the notion that emotional intelligence, particularly optimism, enhances athletic performance. Athletes with higher EI are better equipped to regulate their emotions under pressure, which contributes to greater success (Sukys et al., 2019). Similarly, conscientiousness is often associated with perseverance and better self-regulation, which are critical traits for achieving high performance (Tedesqui and Young, 2020).

The absence of significant differences in other sub-dimensions of EI or conscientiousness across gender and achievement status can be explained by the stable nature of conscientiousness across different populations. Research shows that while conscientiousness influences EI and performance, its variance across demographic groups tends to be minimal (Zhou et al., 2022), suggesting it may have a more generalized impact. In summary, these findings reinforce the role of gender differences in emotional intelligence and the influence of both EI and conscientiousness on athletic achievement. However, conscientiousness remains a stable predictor across varying demographic factors, emphasizing its importance in athletic contexts.

The connection between these two concepts may reflect a reciprocal interaction where conscientiousness can influence emotional responses, contributing to the development of emotional intelligence, and conversely, emotional intelligence can support conscientiousness skills. For instance, an individual with enhanced emotional intelligence may cope more effectively with stressful situations, while an individual with increasing emotional intelligence can strengthen conscientiousness skills, thereby achieving greater emotional balance and success in social relationships. In this way, conscientiousness and emotional intelligence encompass significant cognitive and emotional processes that mutually support and reinforce each other. Future research focusing on experimental applications to determine the emotional intensity of Paralympic athletes could have implications for sports performance and individual personality.

## 5 Conclusion

The study titled “Unveiling the Associations Between Conscientiousness and Emotional Intelligence in Paralympic Athletes “has shed light on the significant relationship between conscientiousness and emotional intelligence (EI) within this unique population. Our findings indicate that emotional intelligence predicts 28% of the variance in conscientiousness among Paralympic athletes. This substantial correlation underscores the critical role of emotional intelligence in fostering self-discipline, perseverance, and a proactive attitude, all of which are vital traits for success in both sports and life.

Moreover, the analysis revealed significant differences in emotional intelligence and conscientiousness based on gender and achievement status. Female athletes tended to score higher in certain dimensions of emotional intelligence, while athletes who had achieved success at the international level demonstrated higher levels of conscientiousness compared to those without international medals. These findings suggest that both gender and achievement status play a role in shaping emotional intelligence and conscientiousness, further highlighting the complexity of these traits in relation to athletic performance.

The results suggest that enhancing emotional intelligence can have a profound impact on athletes’ conscientiousness, potentially leading to improved performance, better coping strategies, and overall psychological well-being. Given the unique challenges faced by Paralympic athletes, such as physical limitations and social stigmas, the development of EI can serve as a crucial component in their holistic development.

### 5.1 Limitations

This study has several limitations that should be considered when interpreting the results. Firstly, the demographic characteristics and specific sports branches of the participants may limit the generalizability of the findings. A key limitation of this study is the use of convenience sampling, which limits the generalizability of the findings to the broader population of Paralympic athletes. While this method allowed for the collection of data from accessible participants, future studies should aim to employ random sampling methods to increase the representativeness of the sample and enhance the external validity of the findings. The diversity of the sports branches represented in the sample may affect the extent to which the results can be applied to all Paralympic athletes. Additionally, the cross-sectional design of the study limits the ability to draw causal conclusions. Longitudinal studies are needed to better understand the causal relationships between conscientiousness and emotional intelligence. Finally, self-reported measures were used to assess both conscientiousness and emotional intelligence, which may introduce bias due to social desirability or inaccurate self-assessment. Future studies should consider using a combination of self-reports, peer-reports, and objective measures to obtain a more comprehensive understanding of these constructs.

### 5.2 Future research

Future research suggests the implementation of targeted training programs focusing on self-awareness, self-regulation, motivation, empathy, and social skills to develop the emotional intelligence (EI) of Paralympic athletes. Coaches should integrate EI development into their methods through workshops and continuous professional development. Regular psychological support and counseling services should be provided to help athletes cope with unique challenges. More longitudinal studies are needed to explore the long-term effects of increased EI on conscientiousness and other life skills. Comprehensive development programs that combine physical training, mental conditioning, and EI enhancement should be developed. Additionally, peer support networks that facilitate the sharing of experiences and strategies for personal growth should be established. By focusing on these recommendations, sports organizations can create an environment that not only enhances athletic performance but also promotes the overall well-being and development of Paralympic athletes, helping them achieve their full potential both on and off the field.

## Data Availability

The original contributions presented in the study are included in the article/supplementary material, further inquiries can be directed to the corresponding author.
